# RNA plasticity and selectivity applicable to therapeutics and novel biosensor development

**DOI:** 10.1111/j.1365-2443.2012.01596.x

**Published:** 2012-05

**Authors:** Yoshikazu Nakamura, Akira Ishiguro, Shin Miyakawa

**Affiliations:** 1 Department of Basic Medical Sciences, University of Tokyo4-6-1 Shirokanedai, Minato-ku, Tokyo 108-8639, Japan; 2 CREST JST, Institute of Medical Science, University of Tokyo4-6-1 Shirokanedai, Minato-ku, Tokyo 108-8639, Japan; 3 Ribomic Incorporation3-16-13 Shirokanedai, Minato-ku, Tokyo 108-0071, Japan

## Abstract

Aptamers are short, single-stranded nucleic acid sequences that are selected *in vitro* from large oligonucleotide libraries based on their high affinity to a target molecule. Hence, aptamers can be thought of as a nucleic acid analog to antibodies. However, several viewpoints hold that the potential of aptamers arises from interesting characteristics that are distinct from, or in some cases, superior to those of antibodies. This review summarizes the recent achievements in aptamer programs developed in our laboratory against basic and therapeutic protein targets. Through these studies, we became aware of the remarkable conformational plasticity and selectivity of RNA, on which the published report has not shed much light even though this is evidently a crucial feature for the strong specificity and affinity of RNA aptamers.

## Introduction

The concept of using single-stranded nucleic acids (aptamers) as affinity molecules for protein or compound binding was initially described in 1990 (Ellington & Szostak 1990, 1992; Tuerk & Gold 1990). The concept is based on the ability of short oligonucleotides to fold, in the presence of a target, into unique three-dimensional (3D) structures that bind the target with high affinity and specificity. Aptamers are generated by a process known as systematic evolution of ligands by exponential enrichment (SELEX), which merges combinatorial chemistry with *in vitro* evolution from a complex library of randomized 10^14−15^ different sequences (Oguro *et al.* 2003; Klussmann 2006; Miyakawa *et al.* 2006, 2008; Ohuchi *et al.* 2006; Keefe & Schaub 2008). Importantly, aptamer targets can be small (e.g., chemical compounds) or large (e.g., proteins), and simple (e.g., purified proteins) or complex (e.g., protein complexes or cell surface receptors). Therefore, aptamers can be used as reagents for affinity purification (Romig *et al.* 1999; Blank *et al.* 2001; Srisawat & Engelke 2001) or as biosensor elements (reviewed in Mairal *et al.* 2008; Mok & Li 2008). Moreover, in December 2004, the US Food and Drug Administration (FDA) approved the first aptamer-based therapeutic, pegaptanib (Macugen), targeting vascular endothelial growth factor for the treatment of age-related macular degeneration (Ng *et al.* 2006; Zhou & Wang 2006).

A characteristic of RNA aptamers is the high potential to create a vast set of tertiary structures, which depend on the different primary sequences. Therefore, it is even likely that some RNA aptamers can fold into structures that resemble protein structures of interest. This idea arose in our previous studies of the structure–function relationship of translation factors, in which we discovered that translation factors mimic the shape of tRNA. One of them, a polypeptide release factor that is required for protein termination, encodes a tripeptide that serves as an ‘anticodon’ to decipher stop codons in mRNA (Ito *et al.* 2000; Nakamura *et al.* 2000). For over four decades, how protein synthesis terminates at stop codons was a long-standing puzzle. The discovery of the ‘peptide anticodon’ undoubtedly solved this persistent coding problem in the genetic code and emphasized a novel concept of molecular mimicry between protein and RNA (Nakamura & Ito 2011).

We speculate that RNA has high potential to create many different tertiary structures, much more than ever thought. The ‘RNA world’ hypothesis (Gesteland *et al.* 1999, 2006) provides the theoretical basis for the potential of RNA to create a variety of tertiary structures. Given this hypothesis, the origin of life was solely made of RNA as multifunctional biomaterials involved in genetic inheritance, cellular architecture and metabolisms; subsequently, the RNA world evolved into the modern ‘DNA/protein world’ by substituting many proteins for the RNA ancestors during the evolution. Therefore, we assume that molecular mimicry might have played an essential role for catalyzing the world transition from ‘RNA’ to ‘protein’. Most of such RNA ancestors have disappeared in the modern DNA/protein world, and we are probably looking at a few molecular fossils that have survived to date in the translation machinery, such as ribosome or tRNA. Nature must have evolved the ‘art’ of molecular mimicry between RNA and proteins using different protein architectures that are functionally active in a ribosome ‘machine’ (Nakamura & Ito 2003). This view reinforces the high potential of RNA for plasticity.

In this review, we present an overview of the structure and function of representative RNA aptamers raised against a variety of human proteins and sensor molecules in our laboratory. This will contribute to our basic understanding of the potential of RNA and the global applications of aptamers.

## Conformational plasticity of RNA as exemplified by anti-IgG aptamer

**Figure 1:**
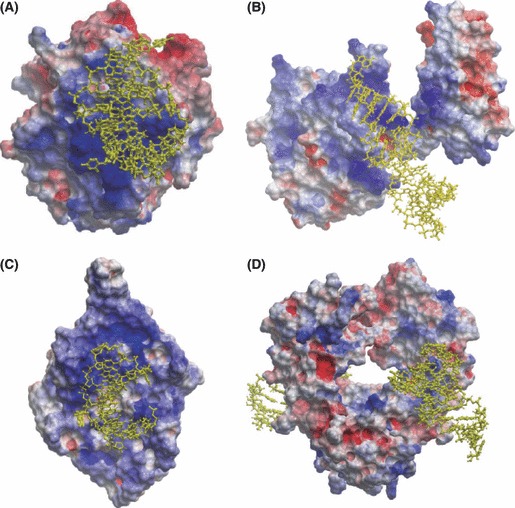
Overall structure of known aptamer–protein complexes with electrostatic surface potential. The RNA aptamer is a yellow ball-and-stick model. (A) Aptamer–thrombin complex at 1.8-Å resolution (Long *et al.* 2008). (B) Aptamer–nuclear factor-κB complex at 2.45-Å resolution (Huang *et al.* 2003). (C) Aptamer–MS2 coat protein complex at 2.8-Å resolution (Horn *et al.* 2004). (D) Aptamer–Fc region of human IgG1 (hFc1) complex at 2.15-Å resolution (Nomura *et al.* 2010). ICM Pro (Molsoft, Inc.) produced images of the electrostatic surface potential using the default setting: The potential scale used was 5. Blue areas: positively charged; red areas: negatively charged.

Although the 3D structures of RNA aptamers are commonly solved by X-ray crystallography or NMR spectroscopy (Hermann & Patel 2000), only three high-resolution structures of RNA aptamers in complex with their targets were reported. These were RNA aptamers in complex with nuclear factor (NF)-κB solved at 2.45 Å (Huang *et al.* 2003), with bacteriophage MS2 capsid solved at 2.8 Å (Horn *et al.* 2004) and with thrombin solved at a resolution of 1.8 Å (Long *et al.* 2008; Fig. 1A–C). NF-κB and the bacteriophage MS2 capsid naturally bind to nucleic acids. The crystal structures of RNA aptamers in complex with the nucleic acid–binding domain of these two proteins reflect these properties by mimicking naturally occurring electrostatic interactions (Ghosh *et al.* 2004; Horn *et al.* 2004). The crystal structure of an RNA aptamer in a complex with thrombin, which is not a nucleic acid–binding protein, indicates that the aptamer binds to the positively charged surface of the protein that is naturally required for high-affinity heparin binding (Long *et al.* 2008). Thus, the crystal structures determined to date have suggested that RNA aptamers bind target proteins predominantly through electrostatic forces (Fig. 1A–C).

**Figure 2:**
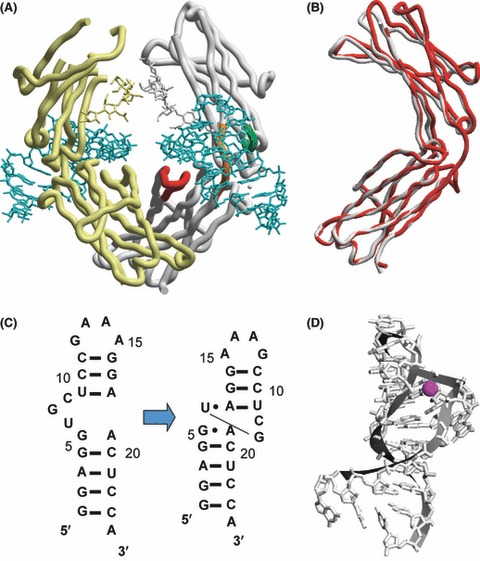
Structure of anti-hFc1 aptamer and the aptamer–hFc1 complex (Nomura *et al.* 2010). (A) The 2.15-Å crystal structure of a human IgG–aptamer complex. hFc1 backbone molecules are light yellow and gray, and bound aptamers are blue. Of the three regions colored red, orange and green in hFc1 (gray), a previous NMR study (Miyakawa *et al.* 2008) suggested that the aptamer binds the orange region, and the crystal structure confirms this prediction. (B) hFc1 conformations uncomplexed (gray) and in complex (red) with the aptamer. (C) *M*-fold-predicted secondary structure of anti-hFc1 aptamer (left) and its crystal structure in the complex (right). The global fold of the aptamer adapts a distorted hairpin structure with base flipping between U6 and G7. (D) Coordination sphere of Ca^2+^ (red sphere). Ca^2+^ is bound in a distorted octahedral coordination environment with the phosphate backbone and five water molecules (Nomura *et al.* 2010).

We selected a 23-nucleotide, high-affinity RNA aptamer against the Fc region of human IgG1 (hFc1; Miyakawa *et al.* 2008). As hFc1 lacks a positively charged protein surface (Deisenhofer 1981), the selected aptamer was speculated to interact with hFc1 via nonelectrostatic forces. The aptamer exhibited remarkable specificity to human IgG and no cross-reactivity to IgGs from other animal species. The aptamer also required divalent cations for binding because the bound IgG was easily released with the addition of EDTA (Miyakawa *et al.* 2008). To investigate these remarkable properties, we solved the crystal structure of the aptamer–hFc1 complex at the resolution of 2.15 Å (Fig. 2A; Nomura *et al.* 2010).

The solved structure showed several interesting features. First, the structure of the aptamer-bound hFc1 was superimposable upon the uncomplexed form of the hFc1 structure (Fig. 2B), indicating that the aptamer binding caused no significant structural changes to the backbone of hFc1. This, in turn, was indicative of the conformational plasticity of RNA to fit the target structure.

Second, the RNA structure in the aptamer–hFc1 complex diverged greatly from the secondary structure predicted by *M*-fold (Fig. 2C). Instead, the structure of the aptamer in complex formed a distorted hairpin structure with base flipping between U6 and G7, producing a GAAA tetraloop, an internal loop, and a terminal A-form helix (Fig. 2C). The internal loop formed by this distorted structure was crucial for binding to hFc1.

Third, the distorted structure was naturally unstable and required the presence of a hydrated calcium ion (Fig. 2D), found in the RNA major groove, that did not coordinate with protein ligands, but bound to nonbridging oxygen atoms of the G7 phosphates in the RNA. Therefore, Ca^2+^ may also help to maintain the distinct conformation of G7, which would be crucial for binding to hFc1. These structural features were consistent with the effect of EDTA, which chelates Ca^2+^, leading to loss of affinity by distorting the aptamer structure. Importantly, affinity was restored upon the addition of Ca^2+^ in the presence of hFc1 (Nomura *et al.* 2010). The reversible binding feature suggested reversible folding of the aptamer, achieved by the presence of the divalent cation and target hFc1.

Protein A affinity chromatography is currently the most frequently used procedure to purify humanized or chimeric antibodies (Fahrner *et al.* 2001; Ghose *et al.* 2005), but also requires an acidic elution step that can sometimes cause unexpected denaturation or inactivation of antibodies (Tsumoto *et al.* 2004; Ghose *et al.* 2005; Cromwell *et al.* 2006). Instead, bound IgGs can be easily released from the aptamer resin under neutral pH conditions using simple elution buffers containing EDTA (Miyakawa *et al.* 2008). Combined with the aptamer’s high specificity to hFc1, these purification advantages provide an alternative reagent for the mass purification of therapeutic antibodies (Miyakawa *et al.* 2008).

Fourth, and most importantly, unlike known RNA–protein interactions, which are generally stabilized by electrostatic forces, as described earlier, the aptamer bound to the neutral portion of the hFc1 surface (Fig. 1D) and the aptamer–hFc1 interaction was stabilized by multiple weak interactions such as hydrogen bonds and van der Waals forces (Nomura *et al.* 2010). For example, the stacking interaction between the aptamer’s G7 and tyrosine 373 (Tyr373) was crucial for the complex; therefore, the aptamer probably interacted through weaker forces supported by van der Waals contacts and hydrogen bonds (Nomura *et al.* 2010). The interaction between hFc1 and the aptamer covered 580 Å^2^ per Fc fragment (Fig. 2A), a surface area that is relatively small compared with that of other RNA aptamer interactions (*c.* 1000 Å^2^), but even so, it achieved remarkably strong affinity (Nomura *et al.* 2010). Therefore, it is likely that SELEX technology can select not only for molecules that interact through predominantly electrostatic forces (Hermann & Patel 2000; Huang *et al.* 2003; Horn *et al.* 2004), but also for high-specificity molecules that interact through weaker forces such as van der Waals contacts and hydrogen bonds. Together, these findings emphasize the excellent conformational plasticity and affinity of RNA molecules, suggesting that RNA aptamers may be applicable to a wider range of targets than previously thought.

## Therapeutic potential of RNA aptamers

On the basis of the conformational plasticity and targeting specificity of RNA, we developed aptamers against various therapeutic target proteins, including cytokines, growth factors, receptors and other regulatory proteins involved in transcription and translation (Oguro *et al.* 2003, 2009; Mori *et al.* 2004; Mochizuki *et al.* 2005; Sakamoto *et al.* 2005; Miyakawa *et al.* 2006, 2008; Tanaka *et al.* 2007; Ohuchi *et al.* 2008; Wang *et al.* 2008; Nakamura *et al.* 2009; Endo & Nakamura 2010; Hiep *et al.* 2010; Adachi *et al.* 2011; Ishiguro *et al.* 2011; Iwagawa *et al.* 2011). Of these, two therapeutic programs approaching clinical trials for autoimmune disorders are described later.

## Therapeutic aptamer against interleukin-17A

Interleukin-17A (IL-17A) is a pro-inflammatory cytokine produced primarily by a subset of CD4^+^ T cells called Th17 cells, which represent a third subset of CD4^+^‘helper’ lymphocytes distinct from the classically described Th1 and Th2 populations (Korn *et al.* 2009; Miossec *et al.* 2009). The primary function of Th17 cells appears to be the clearance of pathogens that are not adequately handled by Th1 or Th2 cells. However, aberrant Th17 responses and IL-17A production have been implicated in a variety of autoimmune diseases and animal models, including rheumatoid arthritis (RA; Chabaud *et al.* 2000; Kirkham *et al.* 2006) and multiple sclerosis (MS; Matusevicius *et al.* 1999; Graber *et al.* 2008).

**Figure 3:**
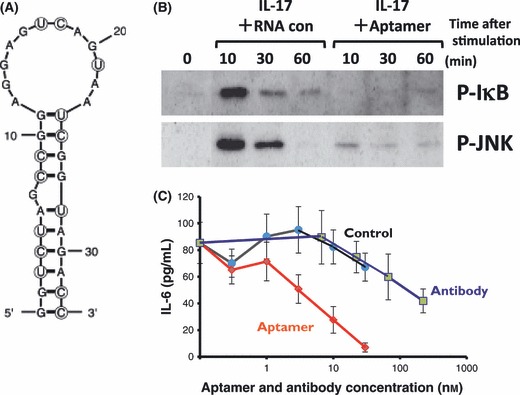
Neutralizing anti-interleukin (IL)-17A aptamer (Ishiguro *et al.* 2011). (A) Secondary structure of Apt21-2, predicted by *M*-fold. Circles denote 2′-fluoro-modified pyrimidines. (B) Suppression of IL-17A-induced signaling pathways in normal human dermal fibroblasts (NHDF cells) by Apt21-2. NHDF cells were treated with human (h)IL-17A (40 ng/mL) with random RNA pool (control) or Apt21-2 RNA (30 nm) and analyzed by Western blotting using the indicated antibodies to detect phosphorylation levels. (C) IL-6 expression affected by Apt21-2 in NHDF cells. hIL-17A was preincubated with Apt21-2 or an anti-hIL-17A antibody at different concentrations and added to NHDF cell culture. After 24-h incubation, the amount of IL-6 secreted to the medium was assessed by ELISA.

To control Th17-based autoimmune diseases, we selected RNA aptamers against human IL-17A (hIL-17A; Ishiguro *et al.* 2011). One such aptamer of 33 nucleotides’ (nts) length, Apt21-2 (Fig. 3A), bound not only to hIL-17A stably, but also to mouse IL-17A (mIL-17A). The dissociation constant (*K*_*d*_) of Apt21-2 to hIL-17A and mIL-17A was estimated to be 48.5 and 701.3 pm, respectively (Ishiguro *et al.* 2011). Importantly, when examined with a sensor chip on which the extracellular domain of IL-17R was fused to Fc and immobilized via protein A, Apt21-2 blocked the binding of hIL-17A to its human receptor hIL-17R as well as of mIL-17A to its mouse receptor mIL-17R (Ishiguro *et al.* 2011). Consistent with this finding, Apt21-2 blocked IL-17A-dependent signaling and hampered phosphorylation of IκB (an NFκB inhibitor) and JNK (*c*-Jun *N*-terminal kinase; Emamaullee *et al.* 2009) proteins in normal human dermal fibroblasts (NHDF; Fig. 3B). Then, we examined the effect of Apt21-2 on the expression of IL-6, one of the cytokines induced by IL-17A, in NHDF cells after 24-hr incubation with hIL-17A. As expected, Apt21-2 definitively blocked the expression of IL-6 in NHDF cells in a dose-dependent manner (Fig. 3C). Of note is that under 1.3-nm hIL-17A conditions, Apt21-2 exhibited an IC_50_ range of 2–3 nm, whereas an available neutralizing anti-hIL-17A monoclonal antibody (mAb317; R&D Systems) had an IC_50_ of 200–300 nm (Fig. 3C). Apt21-2 also inhibited IL-6 production in mouse embryonic fibroblasts (MEF; Molet *et al.* 2001; Dong 2009) induced by mIL-17A, with an IC_50_ of 250–300 nm (Ishiguro *et al.* 2011). Therefore, the efficacy of Apt21-2 is two orders of magnitude greater in human cells than in mouse cells.

**Figure 4:**
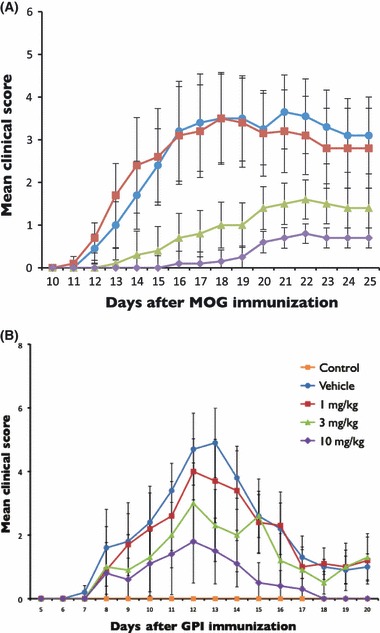
Attenuation of autoimmunity in mouse models by anti-IL-17A aptamer (Ishiguro *et al.* 2011). (A) Suppression of experimental autoimmune encephalitis (EAE) development by Apt21-2 (Ishiguro *et al.* 2011). Wild-type mice (*n* = 10 each) were immunized with myelin oligodendrocyte protein (MOG_35–55_) peptide in complete Freund’s adjuvant, and PEG21-2idT (0, 1, 3 and 10 mg/kg) was administered i.p. every other day after immunization. EAE clinical scores for vehicle and PEG21-2idT–administered mice. Values are the mean and SEM of 10 mice per group. (B) PEG21-2idT treatment suppresses development of glucose-6-phosphate isomerase (GPI)-induced arthritis. DBA/1 mice were immunized with 300 μg of mouse GPI, and the development of arthritis was monitored visually and scored on a scale of 0–2. Values are the mean and SEM of 10 mice per group.

Apt21-2 is composed of 13 ribose 2′-fluoropyrimidines (ribonuclease-resistant) and 20 unmodified purines (Fig. 3A). The pharmacokinetic property of Apt21-2 was further improved by chemical modifications with a 40-kDa polyethylene glycol (PEG) at the 5′ end and an inverted deoxythymidine (idT) at the 3′ end, giving rise to PEG21-2idT. Subsequently, the *in vivo* efficacy of PEG21-2idT was investigated in two mouse models of autoimmunity, experimental autoimmune encephalomyelitis (EAE; Lubberts *et al.* 2001) and glucose-6-phosphate isomerase (GPI)-induced RA (Koenders *et al.* 2005). EAE is a model for the human inflammatory demyelinating disease MS. C57BL/6 mice were immunized with myelin oligodendrocyte glycoprotein (MOG_35–55_) peptide in complete Freund’s adjuvant, and PEG21-2idT was administered intraperitoneally (i.p.; 0, 1, 3 and 10 mg/kg dosages) every other day. The appearance of EAE was significantly delayed in mice administered with 3 and 10 mg/kg PEG21-2idT, with incidence and symptoms reduced markedly in a dose-dependent manner (Fig. 4A). Mice were killed at day 25 and subjected to histological analysis. Consistent with clinical signs, typical foci of MNC infiltration and demyelination were observed in the white matter of the spinal cord of untreated mice, but these signs were not observed in most mice (8/10) administered with 10 mg/kg PEG21-2idT (Ishiguro *et al.* 2011).

Next, GPI-induced RA was induced by immunizing DBA/1 mice with recombinant mouse GPI (mGPI), and 2 PEG21-2idT efficacy tests were conducted. Doses of PEG21-2idT (0, 1, 3, 10 mg/kg) were administered i.p. to DBA/1 mice (*n* = 10) every other day after GPI immunization. PEG21-2idT resulted in significant improvement in the incidence of arthritis and the clinical scores of symptoms in a dose-dependent manner (Fig. 4B). We also injected PEG21-2idT (10 mg/kg, *n* = 10) i.p. every day from day 8 after GPI immunization, a time point at which RA was already established. Importantly, PEG21-2idT injection at day 8 significantly suppressed the progression of arthritis even after it had developed (Ishiguro *et al.* 2011). These results suggest that IL-17A blockade by PEG21-2idT has both protective and therapeutic activity against GPI-induced arthritis.

This study showed that i.p. administration of a PEGylated form of an anti-IL-17A RNA aptamer (PEG21-2idT) inhibits inflammatory lesions and neurological symptoms in EAE and RA mouse models. Although PEG21-2idT was generated against hIL-17, it also exhibited weaker affinity to mIL-17. The intriguing finding in this study relates to the fact that PEG21-2idT was 1–2 orders of magnitude less effective against mIL-17A than it was against hIL-17A, but even so, could exert therapeutic impact in EAE and RA mice. This strongly suggests that the currently generated anti-IL-17A aptamer has potent therapeutic potential for human autoimmune diseases. Such approaches are in progress toward clinical trials.

## Rationalized selection of the aptamer specific to the IL-17A/F heterodimeric form

The IL-17 cytokine family is composed of six structurally related proteins (IL-17A, B, C, D, E and F). Of these, IL-17A and IL-17F are the most closely related to each other, sharing 55% amino acid sequence homology and four conserved cysteine residues at the C-terminal. These conserved cysteine residues participate in the formation of intermolecular disulfide bonds, leading to the formation of homodimeric (IL-17A/A, IL-17F/F) and heterodimeric (IL-17A/F) structures (Chang & Dong 2007; Wright *et al.* 2008). It has been reported that differentiated Th17 cells form IL-17A/F heterodimers in higher amounts than they do either homodimer, and distinct from IL-17F/F, IL-17A/A and IL-17A/F play primary roles in regulating airway inflammation (Liang *et al.* 2007; Wright *et al.* 2007). Furthermore, genetic analyses using IL-17A-deficient and/or IL-17F-deficient mice suggest both overlapping and specific functions for IL-17A and IL-17F (Wright *et al.* 2008; Korn *et al.* 2009). These studies also indicated that IL-17A might be a more important initiating factor than IL-17F in the EAE mouse model or in the development of allergic asthma, whereas both IL-17A and IL-17F contribute to chronic inflammation (Schnyder-Candrian *et al.* 2006; Graber *et al.* 2008). However, the physiological role of IL-17A/F is entirely unknown because of the lack of an experimental system or a reagent that specifically inhibits this heterodimeric form.

**Figure 5:**
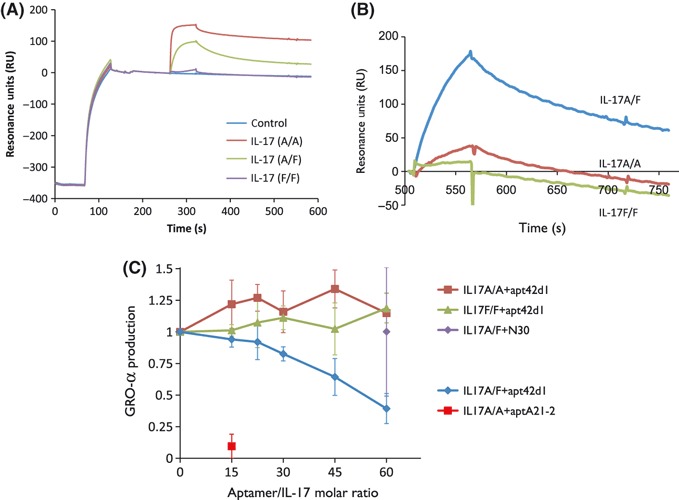
Reactivity of anti-IL-17 aptamers to homo- or heterodimeric forms of IL-17A and IL-17F (Adachi *et al.* 2011). (A) Surface plasmon resonance (SPR) sensorgrams of Apt21-2 injected with homodimeric (IL-17A/A or IL-17F/F) and heterodimeric (IL-17A/F) protein complexes. Poly(A)-tailed Apt21-2 was immobilized to the sensor chip, and IL-17 proteins were injected. (B) SPR sensorgrams demonstrating the affinity of AptAF42 to IL-17A/F, IL-17A/A and IL-17F/F. Poly(A)-tailed AptAF42 was immobilized to the sensor chip, and IL-17 proteins were injected. (C) Suppression of GRO-α production in BJ cells by AptAF42d1. IL-17A/F, IL-17A/A and IL-17F/F were preincubated with the aptamers or N30 RNA (control) at the indicated molar ratios and added to BJ cells. After 6-h incubation in BJ cells, secreted GRO-α was analyzed by ELISA. The *y*-axis denotes the relative amount of GRO-α. The data represent the mean of three independent experiments, and standard deviations are indicated with error bars.

To date, the known anti-IL-17A antibodies react with both IL-17A/A and IL-17A/F dimers. Likewise, Apt21-2 bound to IL-17A/A and IL-17A/F, but not to IL-17F/F in a surface plasmon resonance (SPR) assay (Fig. 5A; Ishiguro *et al.* 2011). We aimed to create an experimental agent that would discriminate IL-17A/F heterodimers from IL-17A/A and IL-17F/F homodimers by SELEX. One such aptamer against human IL-17A/F, AptAF42, was isolated by repeated cycles of selection and counterselection against heterodimeric and homodimeric complexes, respectively. AptAF42 bound to IL-17A/F, but not to IL-17A/A or IL-17F/F (Fig. 5B) and blocked the binding of IL-17A/F, but not of IL-17A/A or IL-17F/F, to the IL-17 receptor in the SPR assay *in vitro* (Adachi *et al.* 2011). Thus, the optimized derivative, AptAF42d1, blocked cytokine GRO-α production induced by IL-17A/F, but not by IL-17A/A or IL-17F/F, in human cells (Fig. 5C). These findings demonstrate that RNA aptamers possess outstanding potential to probe a target structure and that combining selection and counterselection processes enables the selection of a desired aptamer to discriminate heterodimeric from homodimeric structures. Thus, AptAF42d1 is the first inhibitory tool specific to IL-17A/F, which might be applicable to an *in vivo* experiment to elucidate the physiological role of IL-17A/F independent of IL-17A/A and IL-17F/F.

## Therapeutic aptamer against midkine

Midkine (MK) is a heparin-binding growth factor and exerts pleiotropic effects, including cell proliferation, cell migration, angiogenesis and fibrinolysis in a variety of tissues (Muramatsu 2002). MK overexpression has been observed in a number of malignant tumors, Hodgkin’s disease and brain tumors (Muramatsu 2002). However, MK-deficient mice are reportedly resistant to ischemic renal injury (Sato *et al.* 2001) and neointima formation in atherosclerosis (Horiba *et al.* 2000). A recent study proposed that MK deficiency suppresses the development of an RA model by preventing inflammatory leukocyte migration and osteoclast differentiation (Maruyama *et al.* 2004). Furthermore, MK expression in the spinal cord is upregulated during the induction and progression phase of EAE (Liu *et al.* 1998; Hemmer *et al.* 2002). Although MS and EAE have been described as T-helper type 1 (T_H_1) cell–mediated autoimmune diseases, CD4^+^ CD25^+^ regulatory T (T_reg_) cells have recently received a great deal of attention as negative regulators of MS pathogenesis (Kohm *et al.* 2002; Baecher-Allan & Hafler 2004; Viglietta *et al.* 2004; Matarese *et al.* 2005). T_reg_ cells regulate peripheral tolerance and autoimmunity, and abnormalities in T_reg_ cell function may contribute to the development of autoimmune diseases (Sakaguchi 2004, 2005; Liu & Leung 2006). Thus, expansion of the T_reg_ cell population could prevent autoimmune attacks such as gastritis, oophoritis, thyroiditis, inflammatory bowel disease and MS (Kohm *et al.* 2002; von Herrath & Harrison 2003; Mills 2004; Viglietta *et al.* 2004; Matarese *et al.* 2005). This is consistent with the finding that MK-deficient mice are resistant to MOG-induced EAE owing to an expansion of the T_reg_ cell population in the peripheral lymph nodes (Wang *et al.* 2008).

**Figure 6:**
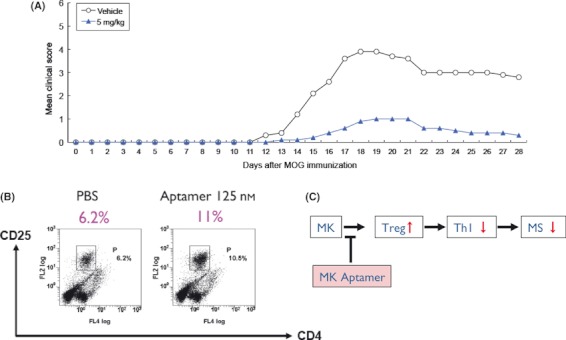
Anti-midkine (MK) aptamer and its therapeutic potential. (A) Clinical scores for wild-type EAE mice administered PBS (*n* = 5) or 5 mg/kg MKapt (*n* = 5) after the MOG injection. (B) Flow cytometric analysis of CD4^+^ CD25^+^ regulatory T (T_reg_) cell population expansion using the anti-MK RNA aptamer *in vitro*. (C) Predicted functional cascade of MK and the anti-MK aptamer.

On the basis of these reports, we isolated RNA aptamers against MK, reflecting a midline’s affinity to heparin; several high-affinity anti-MK aptamers were selected. One such derivative (MKapt) is 38 nts in length and has a *K*_*d*_ of 0.9 nm (Wang *et al.* 2008). MKapt was stabilized by substitutions of ribose 2′-fluoro, omethy, or deoxy nucleotides and modified with cholesterol and idT at the 5′ and 3′ ends, respectively (Ishikawa *et al.* 2008; Wang *et al.* 2008). To investigate the efficacy of the modified MKapt in the pathogenesis of EAE, we immunized C57BL/6 mice with MOG in complete Freund’s adjuvant, and the aptamer was administered i.p. (0 and 5 mg/kg doses) every other day. As shown in Fig. 6A, the appearance of EAE was significantly delayed in mice administered with 5 mg/kg of aptamer, with markedly reduced symptoms. The histological analysis of mice killed at day 28 was consistent with the clinical signs (Wang *et al.* 2008), demonstrating a clear correlation between the clinical and pathological features of EAE in untreated and aptamer-treated mice. Moreover, administration of the aptamer induced expansion of the T_reg_ cell population (Fig. 6B; Wang *et al.* 2008). These findings suggest that MK is a suppressor of T_reg_ cells and that MK blockade by an RNA aptamer may be a potent therapeutic strategy against autoimmune diseases, including MS (Fig. 6C).

## Aptamer-based novel biosensor development

### Anti-Cy3 aptamer

Aptamers have also been generated to dyes and fluorophores such as malachite green (Grate & Wilson 1999) and sulforhodamine B (Holeman *et al.* 1998; Wilson & Szostak 1998). These aptamers are composed of unmodified nucleotides and are thus applicable to sensitive, real-time detection of nucleic acid or small molecules by annealing to complementary sequences or binding to secondary aptamers to target molecules (Kolpashchikov 2003, 2005; Stojanovic & Kolpashchikov 2004). To reduce background fluorescence and increase detection specificity, Kolpashchikov (2005) designed a binary aptamer probe based on the malachite green aptamer (MGA). MGA is unique as it dramatically increases the fluorescence of the dye (Babendure *et al.* 2003). The MGA has a stem–loop structure containing internal bulged loops (Grate & Wilson 1999). These double-stranded sequences were separated into two single-strand sequences, each of which had no affinity to malachite green, and were tagged to sequences complementary to nucleic acid analytes. This probe is referred to as a binary MGA probe and provides immediate fluorescent response after hybridization to complementary nucleic acid analytes, thus offering easy and instant detection of specific DNA and RNA (Kolpashchikov 2005).

The choice of chromophore is crucially important for widespread practical application of aptamer probes to live cell imaging. Although malachite green has been successfully applied to the binary aptamer probe, it remains uncertain whether malachite green is the best choice for intracellular imaging. It is worth mentioning that malachite green very efficiently generates singlet oxygen upon irradiation and is used for targeted damage of mRNA constructs (Grate & Wilson 1999); therefore, it may also lead to undesirable consequences for the behavior of cells during the imaging process. Therefore, an alternative chromophore that is less toxic and better suited for live cell imaging is desirable.

**Figure 7:**
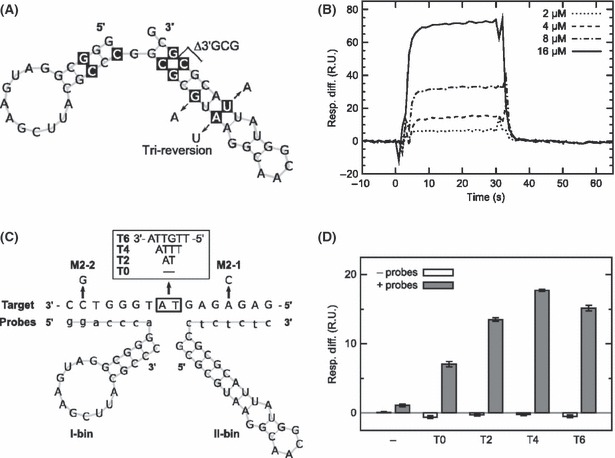
Binary Cy3 aptamer probe composed of folded modules (Endo & Nakamura 2010). (A) Optimized structure of Cy3apt. Ten nucleotides (white letters in black boxes) represent substitutions from the original Cy3apt sequence to optimize affinity to Cy3. ‘Δ3′GCG’ denotes a 3-base deletion on the 3′ end. ‘Tri-reversion’ indicates three bases that reverted to the original. (B) SPR sensorgrams of Cy3apt binding to Cy3 immobilized on the sensor chip. The indicated concentrations of RNAs were injected at time 0 for 30 s at a flow rate of 10 μL/min. (C) Binary Cy3 aptamer probe to detect target oligonucleotides. Schematic representation of the target oligonucleotide and the binary aptamer probe (I-bin and II-bin). The target oligonucleotide T2 sequence is shown, and the variable linker sequences are boxed. M2-1 and M2-2 are single nucleotide mismatches introduced into T2. Target-binding sequences of the binary probe are depicted as lowercase letters. (D) Detection of target oligonucleotides using the binary probe as SPR signals. Target oligonucleotides (10 μm) with (closed box) or without (open box) the binary probe (16 μm) were subjected to SPR analysis.

On the basis of these considerations, we isolated an RNA aptamer against the cyanine dye Cy3 (Cy3apt), a widely used, membrane-permeant and nontoxic fluorophore (Endo & Nakamura 2010). The parental Cy3apt was 83 nts long and shortened to 49 nts length with increased affinity to Cy3 achieved by multiple base changes (Fig. 7A). The affinity of Cy3apt to Cy3 was examined by SPR using a Cy3-immobilized sensor chip injected with different concentrations of Cy3apt. The SPR signal plateaued immediately upon injection of Cy3apt RNA, and plateau levels increased in proportion to the amount of RNA injected, followed by rapid dissociation when the injection stopped (Fig. 7B). Interestingly, the fluorescence intensity of free Cy3 at 580 nm (50-nm bandpass) increased in proportion to the amount of Cy3apt RNA added, with no detectable change in the emission spectra (Endo & Nakamura 2010).

The shortened derivative of Cy3apt is composed of two separate hairpin modules (Fig. 7A). Although each of these domains has no affinity to Cy3 separately, they exhibit affinity to Cy3 upon being properly arranged in a tertiary configuration. Each domain of the Cy3apt was separately used to construct a binary Cy3 aptamer probe. A heptanucleotide, corresponding to each half of a complementary sequence of a 14-nucleotide target sequence (T0 in Fig. 7C), was appended onto the 5′ terminus of one domain and the 3′ terminus of the other domain as a target-binding arm (Fig. 7C; I-bin and II-bin). In contrast to a preceding study (Kolpashchikov 2005), both of our binary probe elements folded into stem–loop structures and had no single-stranded extension except for the appended flanking sequences required for target recognition. The Cy3-binding activity of the binary probe was analyzed using SPR. As shown in Fig. 7D, the designed Cy3 aptamer probe alone did not bind to Cy3 in the absence of the target oligonucleotides. The aptamer probe bound to Cy3 when the target oligonucleotides were present (Fig. 7D). The binding efficiency varied depending on the nucleotide length of the central linker sequences in the target oligonucleotides T2, T4 and T6 (Fig. 7D). The best affinity in these experiments was observed with T4, which contained a tetranucleotide insert, suggesting that the orientation of the two probe elements was important in regenerating the tertiary structure so that it could bind to Cy3. When a single mismatch was introduced into each recognition site of the T2 target sequence (Fig. 7C; M2-1 and M2-2), the binary probe did not bind the target sequence (Fig. 7D), demonstrating single nucleotide discrimination. Unlike the other binary probes consisting of split primary sequences, this binary probe consisted of two folded modules and was referred to as a folded binary probe (Endo & Nakamura 2010).

Sequence-specific detection of nucleic acids is crucial to disease diagnosis, genome study and mRNA monitoring in living cells. Among the numerous nucleic acid analysis methods of particular interest are those that provide immediate visible or fluorescent response after hybridization to complementary nucleic acid analytes, thus offering easy and instant detection of specific DNA and RNA. The binary Cy3 aptamer probe generated in this study will facilitate this approach in view of the fact that it, for the first time, enabled us to deal with a pair of aptamers and Cy3, a commonly used, membrane-permeant, and nontoxic dye, for developing an *in vitro* and *in vivo* sensor system. As the selected aptamer is composed of unmodified (i.e., natural) nucleotides, the *in vivo* sensor system is designable by expressing the binary Cy3 aptamer in test cells and directing its cellular localization, if necessary, using a variety of expression vectors. Moreover, our binary aptamer probe is composed of distinctly folded modules and can be applied to monitor a tertiary RNA–RNA interaction.

### Strategic selection of ‘RNA receptor’ to ‘RNA ligand’

In nature, many RNA molecules and motifs exhibit specific functions that require the formation of a specific 3D structure, rather than simply a linear carrier of genetic code information. The classical examples of such structural, protein-noncoding RNAs (ncRNAs) are tRNA and rRNA, which play key roles in the central dogma of molecular biology (Gesteland *et al.* 1999, 2006). In addition, several regulatory elements on mRNA, such as riboswitches and internal ribosome entry sites, also function via their specific 3D structures (Batey 2006). More recently, several structural ncRNAs have been discovered as specific modulators for intracellular proteins, and it is likely that a significant number of structural RNAs exist within the huge numbers of ncRNAs in higher eukaryote genomes (Mattick & Makunin 2006). Thus, the development of a novel tool to detect and control structural ncRNAs might greatly facilitate genome-encoded ncRNA research, as antibodies did for protein research (Sosnick & Pan 2003; Onoa & Tinoco 2004; Bokinsky & Zhuang 2005; Furtig *et al.* 2007).

**Figure 8:**
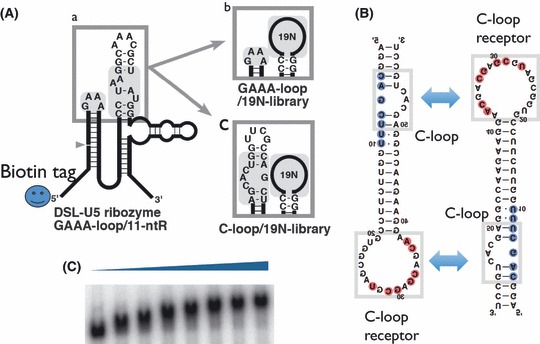
Selection of a novel class of RNA–RNA interaction motifs based on a ligase ribozyme with defined modular architecture (Ohuchi *et al.* 2008). (A) Secondary structures of the parental DSL-U5 ribozyme and its derived libraries. (a) The DSL-U5 ribozyme with the GAAA tetraloop/11-ntR pair essential for the ribozyme activity highlighted in gray. (b) The GAAA loop library with the target GAAA tetraloop and randomized nucleotides highlighted in gray. (c) The C-loop library with the target C-loop motif (C-50) with neighboring single base pairs and randomized nucleotides highlighted in gray. (B) Secondary structure of TectoRNA-derived, homodimer-forming constructs. The target C-loop and the C-loop receptor motifs are enclosed in gray boxes. (C) Autoradiogram of electrophoretic mobility shift assay of the [α-P^32^]-labeled TectoRNA derivative. Left–right: 0, 50, 100, 200, 400, 800 and 1600 nm of unlabeled RNA were added.

To develop such RNA tools, we applied an artificial ligase ribozyme (Designed-and-Selected Ligase, DSL; Ikawa *et al.* 2002) to develop a selection system to generate novel RNA receptor motifs against a target RNA structure within a given structural context (Ohuchi *et al.* 2008). In this system, a GAAA tetraloop and its specific receptor motif (11-ntR) from an artificial RNA ligase ribozyme with modular architecture (the DSL ribozyme) were replaced with a target structure and random sequence, respectively (Fig. 8A). Motifs recognizing the target structure can be identified by *in vitro* selection based on the ribozyme activity. A model selection targeting a GAAA loop successfully identified motifs previously known as GAAA loop receptors (Ohuchi *et al.* 2008). In addition, a new selection targeting a C-loop motif also generated a novel motif, designated ‘C-loop receptor’, which interacts with this structure, although the C-loop is not considered an RNA–RNA interaction motif (Ohuchi *et al.* 2008).

The interaction between the C-loop and the C-loop receptor was investigated by grafting them into TectoRNA, an artificial RNA architecture developed by Jaeger & Leontis (2000) and Atsumi *et al.* (2001), whose self-dimerization properties are suitable for examining the modularity of the selected motif (Fig. 8B). The electrophoretic mobility shift assay showed an apparent reduction in mobility in a concentration-dependent manner (Fig. 8C). The degree of the mobility change was consistent with a biphasic dimerization model with typical fast exchange kinetics, suggesting that the construct dimerized, as was the case for the original TectoRNA (Jaeger *et al.* 2001). Its *K*_*d*_ value, determined as the kinetic equilibrium, was 168 nm (Fig. 8C).

The physical interaction was further investigated by chemical footprinting using a reagent that cleaves the phosphate backbone of RNAs at non-base pairing, solvent-accessible sites (Ohuchi *et al.* 2008). Phosphates around the C-loop motif were cleaved under monomeric conditions but protected under dimeric conditions (Fig. 8B; positions marked blue), indicating that these phosphates, originally located at the surface of the RNA structure, became solvent-inaccessible upon dimerization. This observation supports the physical interaction between the C-loop and the C-loop receptor under dimeric conditions. In contrast, several residues in the C-loop receptor were cleaved efficiently under dimeric conditions but not under monomeric conditions (Fig. 8B; positions marked red). As there was no obvious sequence complementarity between these two sequences, the C-loop receptor is likely to recognize the C-loop motif by specific, non-Watson–Crick tertiary interactions.

Thus, we developed a selection system that enables the identification of novel RNA motifs that interact with a target RNA structure within a desired structural context. We believe that RNA motifs isolated via this selection system can be directly used for RNA engineering, such as the design of artificial RNA architectures (Jaeger & Chworos 2006) or novel molecular tools for desired target RNAs, including structured ncRNAs, regulatory mRNA elements, as well as RNA components of large, complicated ribonucleoprotein complexes such as the ribosome and the spliceosome.

### Cell-based selection of aptamers specific to embryonic stem cell surface markers

Several hundred aptamers have been reported in the published report, and most of them are raised against purified proteins. However, recent development of cell-based SELEX procedures enabled us to isolate aptamers against cell surface molecules of unknown identity or proteins inappropriate for purification in fully active conformations (Morris *et al.* 1998; Ohuchi *et al.* 2006; Shangguan *et al.* 2006; Shamah *et al.* 2008). We have used this procedure to generate aptamers against cell surface markers of unknown identities on embryonic stem cells (ESCs; Iwagawa *et al.* 2011).

Embryonic stem cells are derived from the inner cell mass of blastocysts and able to differentiate into all three germ layers (Evans & Kaufman 1981; Martin 1981; Thomson *et al.* 1998). Under optimized culture conditions, ESCs remain in the undifferentiated state and self-renew indefinitely (Nunomura *et al.* 2005; Watanabe *et al.* 2007; Ying *et al.* 2008; Intoh *et al.* 2009; Shiraki *et al.* 2009; Pera & Tam 2010). Although extensive studies have been pursued to identity cell surface molecules on ESCs (Nunomura *et al.* 2005; Intoh *et al.* 2009) and to uncover the molecular mechanisms underlying the maintenance of the undifferentiated state and regulation of the differentiation process (Chambers & Tomlinson 2009; Young 2011), ESC-specific cell surface proteins, or markers, are not fully understood.

**Figure 9:**
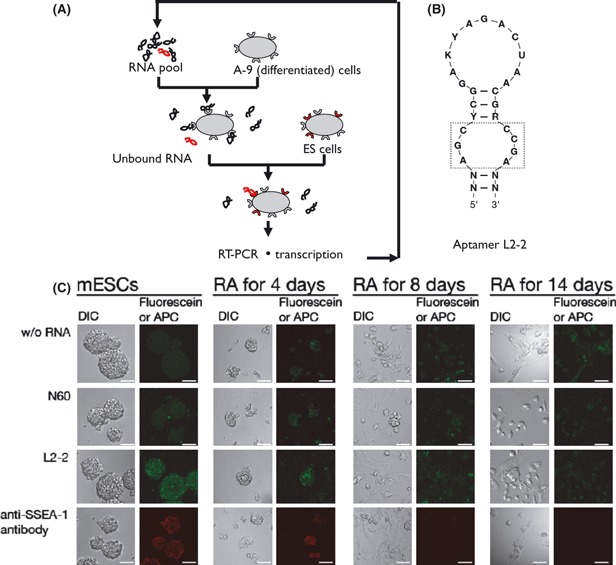
Cell-based selection of RNA aptamer against mouse embryonic stem cells (mESCs; Iwagawa *et al.* 2011). (A) SELEX schematic of live mESCs combined with counterselection against fully differentiated A-9 cells. (B) Consensus motif conserved in the anti-mESC aptamer L2-2. N, K, Y, and R indicate any 4, G or U, C or U, and A or G nucleotides, respectively. (C) Confocal fluorescence image of the L2-2 aptamer. mESCs before and after treatment for rheumatoid arthritis for 4, 8, or 14 days were stained with the indicated fluorescein-labeled RNA probes and APC-labeled antibodies. DIC images are also shown. Scale bar, 50 μm.

Aptamers were selected against intact, live mouse ESCs (mESCs) by SELEX with or without negative selection against fully differentiated A-9 cells (derived from the connective tissues of an adult mouse) from an RNA pool randomized to over 60 nts (N60) with 2′-fluoropyrimidine modifications to resist ribonucleases (Fig. 9A). The bound RNAs were released from the cell surface with a solution containing EDTA to chelate divalent cations. The formation of typical higher-order RNA structures often requires divalent cations, and thus, their elimination with EDTA is expected to inactivate most of the bound aptamers (Pyle 2002; Nomura *et al.* 2010). It is noteworthy that dead and damaged cells tend to adsorb nucleic acids nonspecifically (Raddatz *et al.* 2008), and the elimination of divalent cations rarely affects this nonspecific adsorption. Therefore, EDTA-mediated recovery of aptamers is an effective process to distinguish specific binders from nonspecific adsorbates. L2-2 was one of these aptamers (Fig. 9B).

The binding specificity of [^32^P]-labeled L2-2 was examined against mESCs and five differentiated mouse cell lines: connective tissue (A-9 cells), embryonic fibroblast (NIH 3T3 cells), muscle tissues (C2C12 cells), liver cancer (Hepa 1–6 cells) and neuroblastoma (NB2a cells). The data indicated that this aptamer bound efficiently to mESCs, but failed or only weakly bound, if at all, to the differentiated mouse cell lines (Iwagawa *et al.* 2011). The loss of binding affinity of L2-2 for A-9 cells was consistent with the negative selection process against A-9. Several other mESC-specific aptamers were also isolated in these experiments (Iwagawa *et al.* 2011).

The mESC markers are known to be down-regulated during the course of differentiation (Nash *et al.* 2007; Spencer *et al.* 2007). Confocal microscopy imaging was carried out using fluorescein-labeled L2-2 and mESC-derived differentiated cells by treatment with retinoic acid for 4, 8 and 14 days. The same set of cells was also stained with an antibody against SSEA-1, a canonical mESC marker (Cui *et al.* 2004), as control. A set of fluorescence images showed that aptamer L2-2 as well as anti-SSEA-1 antibody bound to mESCs, whereas the N60 random RNA pool did not (Fig. 9C). Surface staining of the mESCs with the antibody was near absolute, but the staining intensity, or SSEA-1 expression level, differed among individual cells. Similarly, the aptamer staining intensity varied from cell to cell. However, the staining patterns of the antibody and the aptamer were completely different. The aptamer L2-2 preferentially bound to dot-like spots on cell–cell contact regions rather than on the whole cell surface (Fig. 9C). These findings suggest that the L2-2 target is localized on some microdomain structures on the cell–cell contact regions. During the course of retinoic acid–induced differentiation, the staining intensities of anti-SSEA-1 antibody and L2-2 aptamer exhibited different patterns and gradually decreased. The anti-SSEA-1 antibody signal was significantly weakened 4 days after retinoic acid addition and completely disappeared after 8 days (Fig. 9C). In contrast, the reduction of staining with L2-2 proceeded more slowly; a large proportion of the cells were stainable after 4 days, and half of the populations were stainable with the aptamer after 8 days. A few fractions could still be stained with the aptamer even after 14 days.

Given that anti-mESC aptamers could bind to artificially created, induced pluripotent stem cells (iPSCs; Takahashi & Yamanaka 2006; Takahashi *et al.* 2007), these should provide an opportunity for the isolation and purification of iPSCs to evade tumor formation upon transplantation of iPSCs and iPSC-derived cells (Kolossov *et al.* 2006). It has been showed that cell-binding aptamers can be used not only for molecular probes, but also for plating cell adhesion reagents on culture dishes, drug (including short interfering RNA)-delivery systems, *etc*. (Guo *et al.* 2006; McNamara *et al.* 2006; Fang & Tan 2010). Collectively, the anti-mESC aptamer might open the gateway to diverse applications in the fields of regenerative medicine and developmental biology.

## Conclusions and perspectives

In this laboratory, RNA aptamers were selected against a variety of human proteins and a chemical reagent, and the key features were summarized in this review. Although many properties of the selected aptamers were similar to those of antibodies, the aptamers also exhibited superior features. Selected aptamers occasionally had a *K*_*d*_ on the picomolar scale, 100-times stronger affinity than that of normal antibody–antigen interactions. Certain aptamers are more than 50 nts long for specific and high-affinity binding to their target proteins. Occasionally, these proteins lack RNA recognition motifs or an intrinsic, strong affinity to RNA, and the high affinity of the aptamer is achieved through capture of the protein’s global conformation. In contrast, the anti-hFc1 aptamer achieved strong and specific binding to hFc1 mainly by van der Waals contacts and hydrogen bonds rather than via electrostatic forces, unlike most known RNA–protein interactions. These findings demonstrate that RNA has great potential to form a vast set of tertiary structures and to achieve high affinity: We refer to this property as ‘RNA plasticity’. This conformational plasticity and selectivity can be achieved by multiple interactions, which are applicable to many protein targets with low or no affinity to nucleic acids. These results provide us with a solid and promising basis for steps to create novel RNA molecules with distinct structures and with therapeutic potential superior to that of antibodies.

Therapeutic antibodies are being rapidly developed worldwide. In 2010, the US FDA approved 31 therapeutic antibodies, and the antibody therapeutics market is expected to achieve an excess of US$ 40 billion in 2010. Moving forward, aptamer therapeutics is not at a disadvantage. Several characteristics grant aptamers potential superior to that of therapeutic antibodies, including increased binding affinity, *in vitro* manipulation of activity and/or stability, less immunogenicity or toxicity, and scalable chemical production. In contrast to costly cell-based production of antibodies, the production costs of RNA aptamers will also be greatly reduced with the development of oligonucleotide-based therapies. Therefore, RNA aptamers offer a beneficial therapeutic approach for the treatment for diseases not only from a therapeutic standpoint, but also from the perspective of healthcare economics. Table 1 summarizes several aptamers that have undergone clinical trials. The observations that have been made in these trials will provide a better understanding of both the possibilities and limitations of aptamers as therapeutics. Some other applications of aptamers reported in the published report are listed in Table 2.

**Table 1:** Aptamers in clinic

Name	Company	Target	Indication	Phase	References
Macugen	Pfizer/Eyetech	VEGF	AMD	Approved	Ng *et al.* (2006)
AS1411	Antisoma	Nucleolin	Cancer	Phase II	Mongelard & Bouvet (2010) and Bates *et al.* (2009)
REG1	Regado	Factor IXa	ACS	Phase II	Rusconi *et al.* (2002) and Povsic *et al.* (2011)
ARC1779	Archemix	vWF	TTP	Phase II	Jilma-Stohlawetz *et al.* (2011)
NU172	ARCA	Thrombin	CABG	Phase II	Keefe *et al.* (2010)
E10030	Ophthotech	PDGF	AMD	Phase II	Akiyama *et al.* (2006)
ARC1905	Ophthotech	C5	AMD	Phase I	Biesecker *et al.* (1999)
NOX-E36	NOXXON	MCP-1	DN	Phase I	Maasch *et al.* (2008) and Darisipudi *et al.* (2011)
NOX-A12	NOXXON	SDF-1	Cancer	Phase I	Darisipudi *et al.* (2011) and Duda *et al.* (2011)
NOX-H94	NOXXON	Hepcidin	Anemia	Phase I	
BAX499/ARC19499	Baxter/Archemix	TFPI	Hemophilia	Phase I	Waters *et al.* (2011) and Parunov *et al.* (2011)

AMD, age-related macular degeneration; ACS, acute coronary syndrome. TTP, thrombotic thrombocytopenic purpura; CABG, coronary artery bypass grafting; DN, diabetic nephropathy.

**Table 2:** Nontherapeutic applications of aptamers†

Use	Target	Comment	References
*In vivo* imaging	Cancer cell	Activatable probe	Shi *et al.* (2011)
Delivery	PSMA	siRNA conjugate	Dassie *et al.* (2009)
	gp120	siRNA conjugate	Zhou *et al.* (2009)
	E-selectin	Liposome conjugate	Mann *et al.* (2011)
	PSMA	Nanoparticle	Farokhzad *et al.* (2006)
Cell detection/separation	Cancer cell	Microfluidic device	Xu *et al.* (2009)
	Live virus	Virus sensor	Labib *et al.* (2012)
	Cy3	mRNA detection in live cells	Endo & Nakamura (2010)
Somamer	813 proteins	Disease diagnostic	Ostroff *et al.* (2010) and Gold *et al.* (2010)
Aptasensor	K^+^	Beacon/FRET	Kim *et al.* (2012)
	AMP	Electrochemical with [Ru(NH_3_)_6_]^3+^	Shen *et al.* (2007)
	Thrombin	Electrochemical with Methylene blue	Xiao *et al.* (2005)
	IFN-γ	Electrochemical with DNAzyme	Zhang *et al.* (2012)
	IgE	Quartz crystal microbalance	Yao *et al.* (2010)
	Rabbit IgG	Immuno-aptamer PCR	Yoshida *et al.* (2009)
Chromatography	l-selectin	Purification of l-selectin	Romig *et al.* (1999)
	Human IgG-Fc	Therapeutic antibody, Fc-fusion protein	Miyakawa et al. (2008)

† There are many other excellent works that cannot be listed in this table.

To our great surprise, the completion of the Human Genome Project showed the existence of a large amount of ncRNAs. There are two classes of ncRNAs: one that includes antisense RNA and microRNA, and functions by sequence complementarity to target mRNA or DNA, whereas another functions independently of sequence complementarity by forming a functional 3D structure to target protein or macromolecules as an apparent equivalent to a protein. We believe that the former class of ncRNA is likely the tip of the ‘ncRNA iceberg’, regardless of it having received much attention. The latter class of RNAs, which may be referred to as ‘natural aptamers’, might play a crucial role in ncRNA function as well. Therefore, it is important to study both artificial and natural aptamer RNAs to consolidate the superior potential of RNA. The current study of RNA aptamers would be highly beneficial to a comprehensive understanding of genome-coded ncRNA function as well as to the development of RNA medicine.
